# A review article on neuroprotective, immunomodulatory, and anti-inflammatory role of vitamin-D3 in elderly COVID-19 patients

**DOI:** 10.1186/s41983-023-00611-z

**Published:** 2023-02-06

**Authors:** Amit Kumar Tripathi, Sunil Kumar Mishra

**Affiliations:** 1 School of Basic and Applied Science, Galgotias University, Gautam Buddha Nagar, Noida, Uttar Pradesh 203201 India; 2 Department of Pharmaceutical Engineering and Technology, Indian Institute of Technology (Banaras Hindu University), Varanasi, India

**Keywords:** Inflammation, Coronavirus disease 2019, Vitamin D3, Anti-oxidant, Neurological disorder

## Abstract

Vitamin D3 is a secosteroid, broad-spectrum immunomodulatory, antioxidant, and anti-inflammatory hormone produced either by the internal subcutaneous pathway in the presence of ultraviolet B (UVB) rays or by the external pathway in the form of supplements. Vitamin D3 deficiency is a common and reversible contributor to mortality and morbidity among critically ill patients, including Coronavirus Disease 2019 (COVID-19) and other viral infections. The major functions of vitamin D3 are inhibiting the proinflammatory pathways, including nuclear factor kappa B (NF-kB), inflammatory cytokines, such as interleukin-6 (ILs-6), interleukin-18 (ILs-18), and tumour necrosis factor (TNF), preventing the loss of neural sensation in COVID-19, maintaining respiratory homeostasis, and acting as an antiviral, antimalarial, and antihypertensive agent. Vitamin D3 has an important role in reversing the COVID-19 infection in patients who have previously suffered from a neurological disease, such as Alzheimer’s disease, Parkinson disease, motor neuron disease, multiple sclerosis, Creutzfeldt–Jakob disease, stroke, cardiovascular problems, headache, sleep-associated disorder, and others. Moreover, vitamin D3 plays a key role in regulating the gene expression of different pro-inflammatory cytokines. In addition to the information provided above, the current review article provides the most recent information on Vitamin D against COVID-19 with comorbid neurological disorders. Furthermore, we present the most recent advancement and molecular mechanism of action of vitamin D3. Diabetes, cardiovascular disease, and neurological disorders are comorbid conditions, and vitamin D3 is a critical regulator of COVID-19 infection during these conditions. In the midst of the COVID-19 epidemic, factors such as sex, latitudes, nutrition, demography, pollution, and gut microbiota warrants for additional research on vitamin D supplements.

## Introduction

**Fig. 1:**
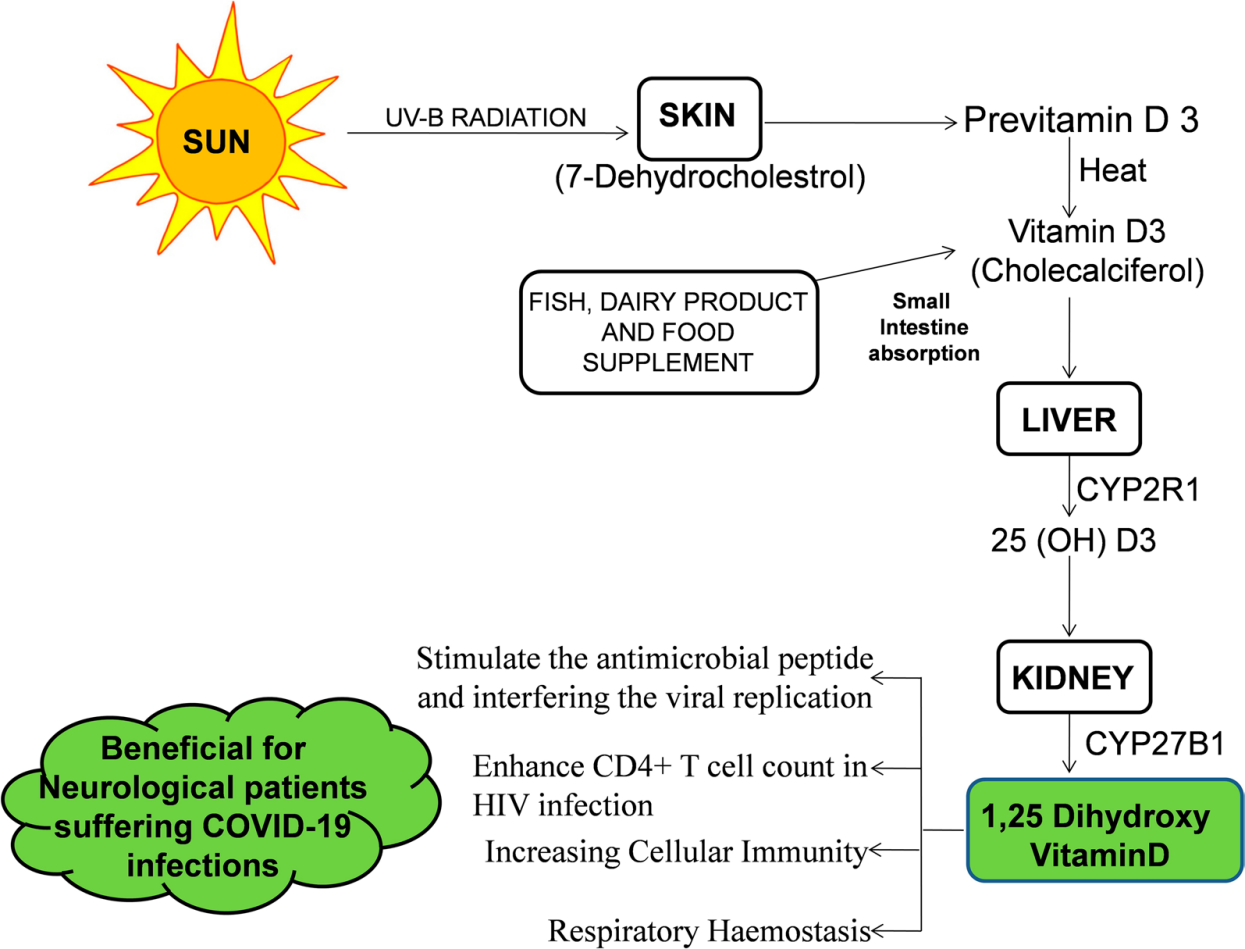
Endogenously production of Vitamin D3 in human skin, liver, and kidney from UV-B radiation from sunlight and exogenously supplied by food supplements is depicted schematically. Vitamin D3 plays a very crucial role in different types of viral infections, including in COVID-19 patients with many neurological diseases, including headache, insomnia, and stroke. Vitamin D3 improves immunity in immunocompromised, neurologically impaired COVID-19 patients suffering from hyperinflammation, thrombosis, and oxidative stress

Vitamin D3 is an anti-inflammatory, and anti-oxidant with a broken ring of secosteroid hormone produced internally with the effect of UV-B radiation (Fig. 1) on the skin or as an externally available food supplement [1–5].The shortage of Vitamin D3 is a public health problem affecting billions of people worldwide. Several studies have found a link between vitamin D3 deficiency and coronavirus disease 2019 (COVID-19) (Table 1) infections [4]. Given the massive health and economic burden of COVID-19, any option to accelerate and improve health and reduce the risk of health degeneration and death would be economically and clinically significant. The major organ affected by COVID-19 is the lung, which is a major respiratory organ, and the maximum number of angiotensin converting enzyme 2 (ACE2) receptors are present on this organ. The respiratory tract alveoli of COVID-19 patients bear ACE2 receptors, which have a tendency to bind with the spike protein of the SARS-CoV-2 virus, enter the host cell. At early stages of inflammation, a protective immune response is accountable for removing the virus, and therefore, strategies to improve the immune responses are important [1]. As the disease progresses, lung inflammation occurs due to the release of several proinflammatory cytokines, such as interleukin (IL)-1B, IL-18, and IL-1 by activated macrophages and type 1T helper (Th1) immune cells. However, elderly comorbid patients suffering from different neurological problems who are immunocompromised are at higher risk of infection from COVID-19 [6–10]. Unfortunately, the current knowledge gap on the potential role of vitamin D3 in neurological patients [5] suffering with headache and sleepless anomalies during the COVID-19 infections is unmet need to deciphered; however, the potential antioxidant vitamins D alleviate the severity and improve the outcomes in these patients. Vitamin D3 insufficiency is also associated with diabetic neuropathy [11], Peripheral neuropathy, COVID-19, in age-related neurodegenerative diseases [12]. Among environmental factors, seasonal variation in sun exposure, UV exposure, geographic latitudes, air pollution, age, and sex–gender differences, pregnancy in high latitudes, obesity, and darker skin all affect endogenous Vitamin D3 formation by sunlight [13, 14].

**Table 1:** Various studies conducted for the beneficial and neuroprotective role of Vitamin D3

Major remark	COVID-19 cases	Hospital/countries	References
Vitamin D3 (1000 IU), Mg (150 mg), and vitamin B12 (500 µg)	43	Singapore	[2]
No major outcomes due less sample	92	USA	[41]
Hypo-vitaminosis D in COVID-19 Patients	137	Italy	[42]
1000 IU (25 mg) per day	Variable according the region	UK, Iceland, Norway, Finland	[43]

### Molecular mechanism of action of vitamin D3 against COVID-19

**Fig. 2:**
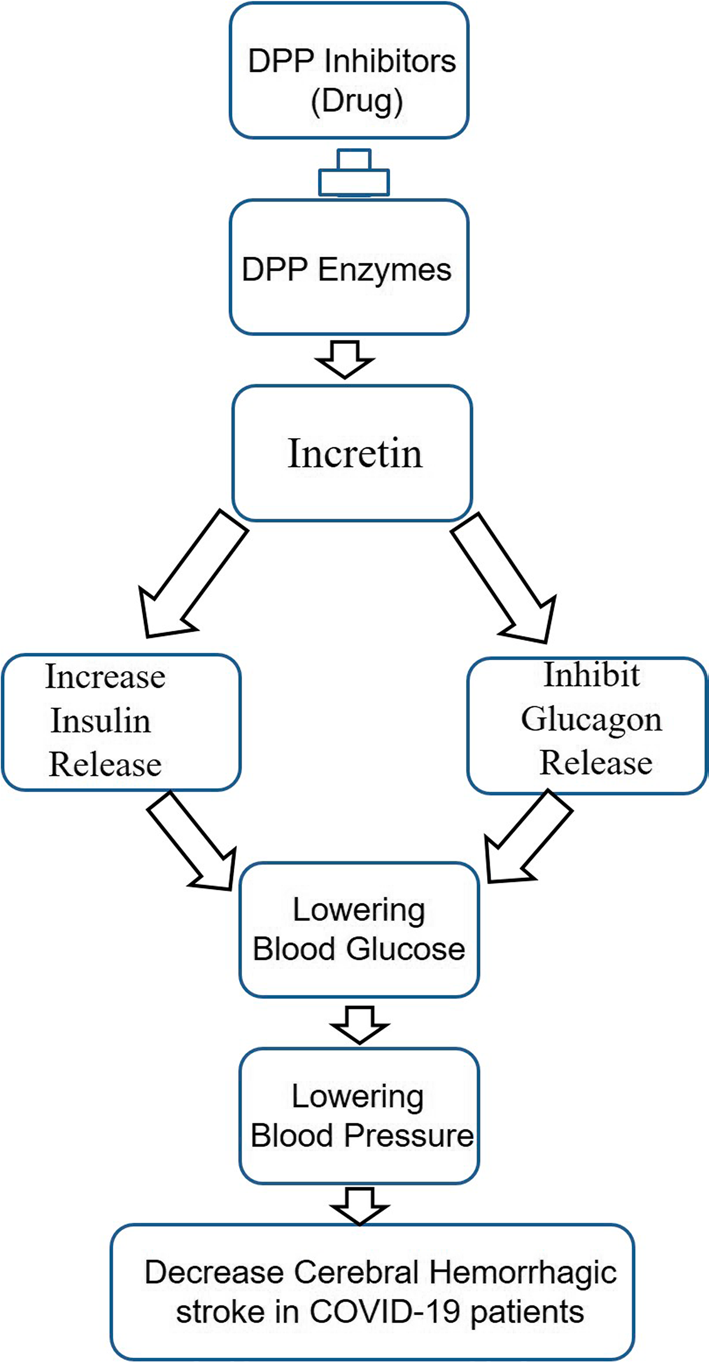
Figure 2 shows the role of dipeptidyl peptidase-4 (DPP-4) inhibitors, which inhibit the DPP-4 enzymes, which down-regulate the incretin. DPP-4 inhibitors are showing neuroprotective potential in synergistic combination with Vitamin D in COVID-19 patients suffering from neurological disorders. Incretins are a group of metabolic hormones that stimulate insulin and inhibit glucagon, which finally decreases the blood glucose level and pressure and ultimately reduces the chance of cerebral hemorrhagic stroke in COVID-19 patients

Cytokine storm (hyperinflammation) plays a central role in the pathophysiology of COVID-19, caused by severe acute respiratory syndrome coronavirus disease 2 (SARS-CoV-2). A dipeptidyl peptidase-4 (DPP-4) inhibitor is a potential candidate for downregulating the hyperinflammation in COVID-19 patients[15]. DPP-4 is the enzyme that inactivates the hormone incretin, which enhances insulin production when it is needed. DPP-4 inhibitors (DPP-4i) are a class of anti-hyperglycemic drugs used for the treatment of type-2 diabetes (T2D) and can better repurposing drug for COVID-19 patients (Fig. 2). Both Vitamin D3 and DPP-4i exert synergistic way for anti-inflammatory and immunomodulatory [16, 17]. Vitamin D3 biologically active metabolites 1,25-dihydroxyvitamin D ((1,25(OH)2D) is able to downregulates the nuclear factor kappaB (NF-κB)-dependent pro-inflammatory cytokine secretion, such as IL-6 and IL-2, by blocking NF-κB p65 activation via up-regulation of the NF-κB inhibitory protein IkB-α [18]. Vitamin D3 is capable of regulating gene expression, which results in an increased innate immune response and a lowered acquired immune response. The major innate immune responses have increased nitric oxide release, lysosomal enzymes, Toll-like receptors (TLR) expression, and beta-defensin. The expression of the DPP-4 receptor is reduced significantly in vivo upon the sufficient supplementation of vitamin D3 [19].

Recent trials have reported that vitamin D3 has a non-classical role in reducing lethal pneumonia in COVID-19 and other respiratory tract infections (Table 1) [3, 20]. Several studies determined that there is a negative correlation between Vitamin D3 and COVID-19 cases per million people in European countries [4]. A recent report suggested that Vitamin D3 plays an inhibitory role in viral DNA replication with its anti-inflammatory and immunomodulatory effects [21].

### Vitamin-D, COVID-19 and neurological impairment

There is an unmet need to establish the relationship between the COVID-19 pandemic and neurological disorders. COVID-19 is associated with several neurological disorders, such as headache, sleep-associated disorder, stroke, motor neuron disease, alzheimer disease, parkinson disease, multiple sclerosis, and Creutzfeldt–Jakob disease [7, 10, 22–25]. Bilateral and long-lasting headaches are most common in COVID-19 patients [22]. The three major characteristics of headaches during the pandemic are duration, severity, and frequency. Headache is the central and most significant second tier of symptoms of COVID-19 pandemic, and the most common symptoms are long COVID-19 headaches and post-vaccination headaches [26, 27]. The timing of post-vaccination headache in subjects with or without an associated cerebrovascular accident occurs within 1 day and is rarely associated with a cerebrovascular accident [26]. A delayed headache 3 days after vaccination was the accurate diagnostic biomarker for a cerebrovascular accident [26]. The headache occurs in 55.5% of the COVID-19 patients. Female gender, fever, dehydration, primary headache and decreased platelet counts are the common predators of COVID-19 [28]. is associated with a higher prevalence of headache during COVID-19 [29]. Another study reported that Vitamin D3 deficiency in a certain group of children is related to decreased sleep duration and less sleep efficiency [23]. Patients with dementia should receive extra care and close monitoring to reduce the risk of death from COVID-19 infections [24]. Large vessel acute ischemic stroke with worse functional outcomes and more mortality is most common in COVID-19 compared to non-COVID-19 patients [30]. Mild COVID-19 infection may be a major cause of macro-thrombosis, cardiovascular, and stroke-related incidents [31]. COVID-19 patients who are advised to take daily Vitamin D3 supplementation (2000–5000 IU/day) in older parkinson disease patients have the potential to slow parkinson disease progression and reduce COVID-19 infections [5]. Supplementation of Vitamin D3 with Mg and Zn has the potential to minimize the three risk factors such as hyperglycemia, hypertension, and hyperinsulinemia that increase the effects of inflammation, and thrombosis [28]. The above statement was supported by the studies that suggested that 25-hydroxyvitamin D3 (41.19 nmol/L) levels protect against COVID-19 infections [32]. Because of elevated blood pressure and altered cerebrovascular endothelial function, hemorrhagic stroke is more common in COVID-19 patients than ischemic stroke [6, 33]. DPP inhibitors drug are also responsible for downregulating the blood pressure and beneficial for COVID-19 patients to reduce the chance of hemorrhagic stroke (Fig. 2). Anticoagulation therapy may be considered with patients with COVID-19, though the risk of Intracerebral hemorrhage in the treatment regimen [33]. According to the findings of the study, intracerebral haemorrhage (11.6%) occurred less frequently in the 108,571 patients with COVID-19 who had acute cardiovascular disease (1.4%) or ischemic stroke (87.4%) [31]. Another study suggested that the average age of stroke in COVID-19 patients is 65.5 and deranges clinical parameters, such as the coagulation profile, liver function test, and full blood counts [34]. Around 200 Italian neurologists sent the report from about 90 multiple sclerosis reports across Italy, claiming that depression and anxiety are the most common features of multiple sclerosis patients suffering from COVID-19 [35]. Creutzfeldt–Jakob disease is a prion disease that accelerates inflammatory pathogenesis in COVID-19 patients [36]. The vulnerability of SARS-CoV-2 and COVID-19 is greater among neurological patients, suggesting an urgent need for the development of suitable medications, vitamins, and dietary supplements. Vitamin B2 is an important neuroprotective molecule against cerebral stroke via c-jun signaling pathways [37]. Many phytochemicals, including piperine, a re-purposing drug, play an important role in COVID-19 by inhibiting the receptor [38]. Plenty of studies have found that age and neuropathology have different determinants in neurodegenerative disease, such as age in Parkinson's disease and neuropathology in MS. Vitamin D binding protein-1 (DBP-1) polymorphism (DBP-1, 2, and 3 phenotypes) suggested that COVID-19 infections are associated with DBP-1 frequency [39, 40]. The support for a 30% DBP reduction in COVID-19 patients indicates the need for more focused research on COVID-19-related vitamin D insufficiency and DBP therapeutic target validation [32].

## Conclusion

The COVID-19 patients with neurological manifestations, vitamin D supplementation play a key role in accelerated recovery from the infection. Prevalence of vitamin D deficiency in serum of adult and elderly COVID-19 patients increase the mortality and hospitalization from COVID-19. However, incidence of the COVID-19 infection is more common in countries that receive moderate sunlight duration, which is a major concern for the prevention and management of COVID-19 infections. To combate these situation there is geographic migration of population during the current pandemic can be affected by demographic characteristics, latitudes and pollution. Population and urbanization play a very important role for the insufficiency in vitamin D during pandemic. However, food supplements such as fish oil, dairy products, and others can increase the level of vitamin D3 in an elderly population. COVID-19-fighting strategies also include the dietary intake of vitamins (Vitamin C and Vitamin B12), and minerals and improved gut microbiota diversity by dietary intake of phytochemicals and moving to different geographical locations with enough UV-B radiation to accelerate endogenous Vitamin D3 synthesis. The exogenous supplementation of the Vitamin D3 (250,000–500,000 IU) is safe for ventilated patients and those with severe neurological impairment. Vitamin D3 along with other vaccines and appropriate COVID behavior would be beneficial for future any viral infections. Randomized controlled clinical trials and large-scale cohort studies, and vitamin D supplementation has protective measure against COVID-19 infections.

## Data Availability

Data supporting results found in manuscript.

## Abbreviations

COVID-19: Coronavirus disease-2019
VDRE: Vitamin D3 receptor element
DPP-4: Dipeptidyl peptidase-4
VIDDPP-4i: Vitamin D3 and DPP-4 inhibitor
UV-B: Ultraviolet-B
